# IgG sialylation occurs in B cells pre antibody secretion

**DOI:** 10.3389/fimmu.2024.1402000

**Published:** 2024-05-17

**Authors:** Anja Werner, Maja Hanić, Olga O. Zaitseva, Gordan Lauc, Anja Lux, Lars Nitschke, Falk Nimmerjahn

**Affiliations:** ^1^ Department of Biology, Division of Genetics, Friedrich-Alexander-Universität Erlangen-Nürnberg, Erlangen, Germany; ^2^ Genos Ltd, Glycoscience Research Laboratory, Zagreb, Croatia; ^3^ Medical Immunology Campus Erlangen, Friedrich-Alexander-Universität Erlangen-Nürnberg, Erlangen, Germany

**Keywords:** immunoglobulin G, sialic acid, autoantibody, B cell, sialylation

## Abstract

Sialic acids as terminal sugar residues on cell surface or secreted proteins have many functional roles. In particular, the presence or absence of α2,6-linked sialic acid residues at the immunoglobulin G (IgG) Fc fragment can switch IgG effector functions from pro- to anti-inflammatory activity. IgG glycosylation is considered to take place inside the plasma blast/plasma cell while the molecule travels through the endoplasmic reticulum and Golgi apparatus before being secreted. However, more recent studies have suggested that IgG sialylation may occur predominantly post-antibody secretion. To what extent this extracellular IgG sialylation process contributes to overall IgG sialylation remains unclear, however. By generating bone marrow chimeric mice with a B cell-specific deletion of ST6Gal1, the key enzyme required for IgG sialylation, we now show that sialylation of the IgG Fc fragment exclusively occurs within B cells pre-IgG secretion. We further demonstrate that B cells expressing ST6Gal1 have a developmental advantage over B cells lacking ST6Gal1 expression and thus dominate the plasma cell pool and the resulting serum IgG population in mouse models in which both ST6Gal1-sufficient and -deficient B cells are present.

## 1 Introduction

Immunoglobulin G (IgG) antibodies are an essential component of humoral immunity. Binding of IgG to Fcγ receptors (FcγRs) can lead to the release of pro-inflammatory mediators, phagocytosis, or antibody-dependent cellular cytotoxicity (ADCC) (1, 2). Besides the important role of IgG molecules in the elimination of pathogenic microorganisms, self-reactive IgG antibodies are responsible for tissue destruction and inflammation during autoimmune diseases such as primary Sjögren’s syndrome (pSS), systemic lupus erythematosus (SLE), or rheumatoid arthritis (RA) (3, 4). Of note, pooled serum IgG preparations from thousands of donors, called IVIg (intravenous immunoglobulin G), are used as an anti-inflammatory treatment for several autoimmune diseases and chronic inflammation, suggesting that IgG can also have immunomodulatory functions (5). Several studies have shown that at least some of these immunomodulatory functions of IgG depend on IgG glycosylation. Each IgG molecule contains two bi-antennary sugar moieties attached to each of the conserved asparagine (N) 297 residues in the IgG constant domain 2 (CH2). This sugar structure consists of a constant core structure of N-acetylglucosamine and mannose residues and variable additions of penultimate fucose or terminal galactose and sialic acid residues. In addition to this, N297-linked sugar moiety IgG molecules may gain additional glycosylation sites in the fragment antigen binding [F(ab)] region during the process of somatic hypermutation and affinity maturation (6). In the serum IgG pool, roughly 15%–25% of IgG molecules contain F(ab)-associated N-linked sugar domains (7). More recent studies suggest that anti-citrullinated protein antibodies, which highly correlate with the development of rheumatoid arthritis, are heavily F(ab)-glycosylated, indicative of highly specific antigen-driven selection processes (8–10).

In contrast to the fully processed sugar moiety of the IgG F(ab) domain, the Fc-linked sugar moiety only contains minor levels of terminal sialic acid residues. This is at least in part due to the fact that the Fc-linked sugar moiety is buried in the hydrophobic pocket between the two Fc domains (11). Functionally, the Fc-linked sugar moiety and especially terminal sialic acid residues have been shown to contribute to the therapeutic activity of IVIg in a variety of murine model systems of autoantibody-driven inflammation (12, 13). Moreover, patients with active autoimmune diseases like RA show a reduction of IgG galactosylation and sialylation during the acute disease (14). Of note, IVIg preparations enriched for IgG glycovariants rich in sialic acids via *Sambucus nigra* agglutinin (SNA) enhance the anti-inflammatory activity of IVIg, opening a therapeutic avenue to increase IVIg activity or to generate recombinant IVIg replacements at least for specific indications (12, 15, 16).

Because of this important role of glycosylation for fine-tuning IgG effector functions, an in-depth understanding of how B cells glycosylate IgG is required. Traditionally, IgG glycosylation is considered to take place inside the plasma cell or plasma blast before secretion. While the core sugar moiety is attached to the IgG molecule in the endoplasmic reticulum, a remodeling of this core structure occurs during the transport through the cis-, medial-, and trans-Golgi, resulting in the mature sugar structure observed on IgG antibodies present in the serum. The sugar donors and N-glycan precursors required for this process are generated in the nucleus, lysosomes, or the cytoplasm (17). In this context, α2,6-linked sialic acid residues can be attached to exposed galactose residues selectively through the action of a single sialyltransferase, ST6Gal1 (18). ST6Gal1 is expressed in hematopoietic cells, as well as in liver cells and catalyzes the addition of sialic acids via an α2,6-linkage to the non-reducing end of terminal lactosamine structures (19, 20). By inactivating the ST6Gal1 gene in mice, all α2,6-linked sialic acids on IgG as well as on all other sialylated glycoproteins in the body are eliminated (21).

However, this current view on IgG sialylation was challenged more recently, and it was suggested that terminal α2,6-linked sialic acids may be attached independently of the B-cell secretory pathway post-antibody secretion from plasma cells/plasma blasts (22). This, however, raises major concerns for the use of therapeutic antibodies as this mechanism could allow therapeutic antibodies to change their glycosylation status post-injection into the patient. Indeed, several studies suggested that highly sialylated cytotoxic antibodies may have an altered binding to FcγRs and thus change their activity. We have recently addressed to what extent injected IgG antibodies change their sialylation profile during the first week upon injection into mice. While we were able to show that a very small fraction of terminal sialic acid residues may be acquired post-IgG secretion in the more accessible F(ab)-linked sugar moieties, we have not been able to detect *de novo* sialylation of the terminal galactose residues present in the N297 Fc-linked sugar moiety. Whether a further increase in IgG sialylation may occur at later time points remained open, however.

In the current study, we further extend our findings and demonstrate that IgG sialylation occurs predominantly if not exclusively in plasma cells/plasma blasts before secretion of antibodies into the serum. By generating different bone marrow (BM) chimeric mice in which ST6Gal1 is exclusively lacking in B cells, we demonstrate that serum IgG sialylation is absent despite the presence of ST6Gal1 in all other immune and non-immune cell subsets (23). We further show that very low amounts of residual SNA^+^ plasma blasts and plasma cells that escape deletion in either genetic or BM irradiation chimeric mice have a selective survival advantage and thus can dramatically impact IgG sialylation in the serum.

## 2 Results

### 2.1 Impaired development of mature B-cell subsets in ST6Gal1^−/−^ mice

**Figure 1:**
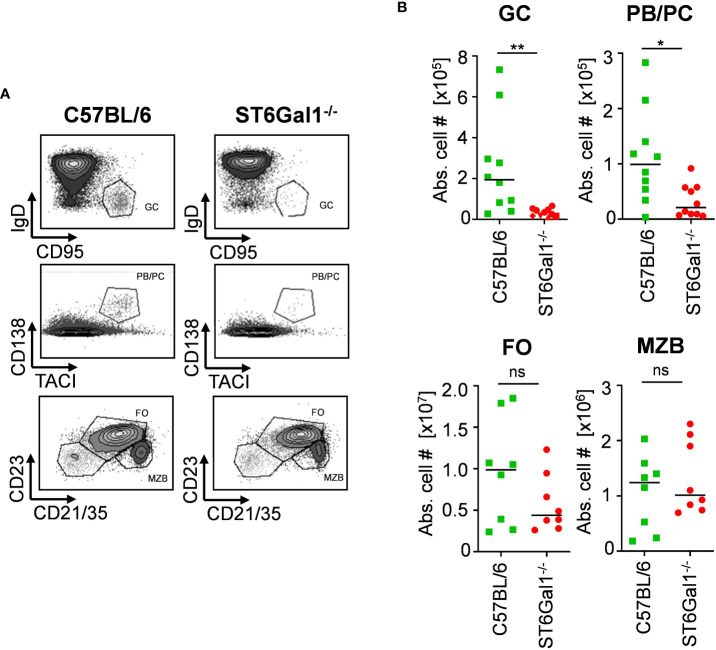
Impaired B-cell development in ST6Gal1^−/−^ mice. Germinal center B cells (GC; B220^+^ CD95^+^ IgD^−^), plasma blasts/plasma cells (PB/PC; CD138^+^ TACI^+^), follicular B cells (FO; B220^+^ CD21/35^+^ CD23^+^), and marginal zone B cells (MZB; B220^+^ CD21/35^high^ CD23^int^) in the spleen were identified via flow cytometry. **(A)** Representative dot plots and **(B)** quantified absolute cell numbers (abs. cell #) of the indicated B-cell subpopulations per spleen are shown. Normal distribution was tested by Shapiro–Wilk test, and significant differences between indicated groups were determined with unpaired *t*-test. *p < 0.05; **p < 0.01; ns, not significant.

One major concern with using genetic model systems, such as the Cre-flox system, to delete target genes in a cell subset-specific manner is that the deletion is not complete. This, in turn, may lead to a competitive developmental advantage of cells that have escaped deletion, if the lack of expression of the gene of interest impacts cell proliferation, development, or survival, for example (24, 25). To investigate if the lack of α2,6-linked sialic acid residues leads to altered B-cell development, we investigated different B-cell populations in total ST6Gal1^−/−^ knockout mice. By analyzing splenic B-cell compartments via flow cytometry (gating strategy, see **Supplementary Figure 1A**), we found reduced numbers of germinal center B cells (B220^+^, CD95^+^, and IgD^−^) and plasma blasts and plasma cells (CD138^+^ and TACI^+^) in ST6Gal1-deficient mice, consistent with previous reports (26, 27). However, other B-cell subpopulations like follicular and marginal zone B cells were not significantly reduced (**Figures 1A, B**). Thus, B cells lacking ST6Gal1 expression indeed have an impaired development into activated or antibody producing B-cell subsets and thus may have a competitive disadvantage in mice in which wild-type and ST6Gal1-deficient B cells co-exist. However, mechanistical studies regarding the role of α2,6-linked sialic acids in B-cell development are currently under investigation and beyond the scope of this study.

### 2.2 Generation of mice with a B cell-specific deletion of ST6Gal1

**Figure 2:**
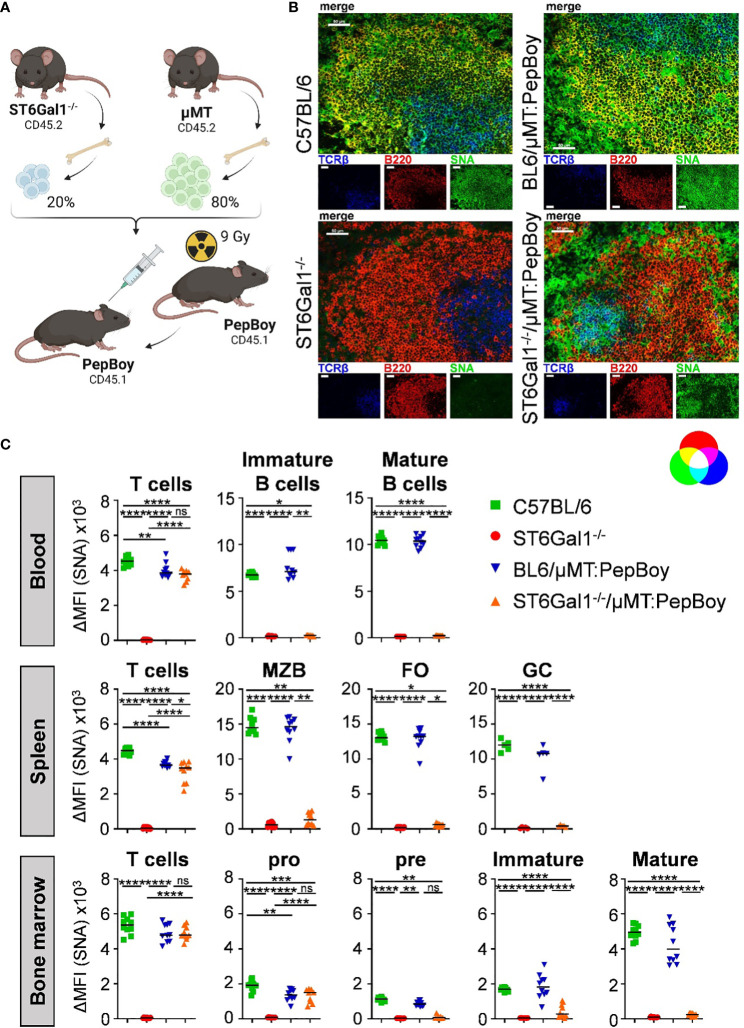
ST6Gal1^−/−^/µMT: PepBoy mice lack sialic acids specifically on B cells. **(A)** Shown is a schematic representation of the experimental setup. ST6Gal1^−/−^/μMT: PepBoy mice were generated by sublethally irradiating 6-week-old B6.SJL-Ptprc^a^Pepc^b^/BoyJ (PepBoy) mice with 9 Gray (Gy) and reconstitution with 20% bone marrow of ST6Gal1^−/−^ mice and 80% bone marrow of μMT mice. Control groups were C57BL/6, ST6Gal1^−/−^ and BL6/μMT: PepBoy mice receiving 20% bone marrow of C57BL/6 mice instead of ST6Gal1^−/−^ mice. **(B)** Representative immunofluorescence images of spleen sections stained with SNA in combination with the indicated cell lineage antibodies identifying B cells or T cells are shown. White bars correspond to 50 µm. **(C)** Detection of SNA expression on B- and T-cell subsets via flow cytometry. TCRβ^+^ T cells and B-cell subpopulations of blood, spleen, and bone marrow were analyzed by flow cytometry 8 to 10 weeks after reconstitution. B-cell subpopulations of indicated organs were distinguished by their specific surface marker expression: in the blood, mature (B220^+^ IgM^+^ IgD^+^) and immature B cells (B220^+^ IgM^+^ IgD^−^) were distinguished. In the spleen, B cells were divided into marginal zone B cells (MZB; B220^+^ CD23^int^ CD21/CD35^high^), follicular B cells (FO; B220^+^ CD23^+^ CD21/CD35^+^), and germinal center B cells (GC; B220^+^ IgD^−^ CD95^+^). B-cell populations identified in the bone marrow were pro (B220^+^ IgM^-^ CD43^+^), pre (B220^+^ IgM^-^ CD43^-^), immature (B220^+^ IgM^+^), and mature (B220^high^ IgM^+^) B cells. Depicted are delta median fluorescence intensities (ΔMFI) of stainings with and without SNA. *p < 0.05; **p < 0.01; ***p < 0.001; ****p < 0.0001; ns, not significant.

To generate a suitable mouse model for studying if α2,6-linked sialic acids are added pre- or post-IgG secretion and to circumvent potential issues with classical Cre-flox systems, we made use of a BM chimeric approach, which has been used successfully before to create mice with a B cell-specific deletion of target genes (23). For this, we generated BM chimeric mice using sublethally irradiated wild-type mice (PepBoy mice expressing CD45.1) as BM recipients and a BM cell mixture, consisting of 20% BM of ST6Gal1^−/−^ mice and 80% BM of B cell-deficient, ST6Gal1^+/+^ µMT BM donor mice (both CD45.2) (**Figure 2A**). This generates mice that lack ST6Gal1 specifically in B cells as µMT mice lack mature B cells but can generate all other immune cell subsets (ST6Gal1^−/−^/μMT: PepBoy mice). As controls, C57BL/6, total ST6Gal1^−/−^ mice, and BM chimeric mice receiving a mixture of 20% BM of C57BL/6 and 80% BM of µMT mice (BL6/μMT: PepBoy mice), which have a normal expression of ST6Gal1 on B cells, were included.

By using immunofluorescence staining of spleen sections with SNA, which specifically detects α2,6-linked sialic acid residues in combination with lineage markers for B cells and T cells, we could confirm the absence of sialic acids on B220^+^ B cells in ST6Gal1^−/−^/μMT: PepBoy mice. In contrast, TCRβ^+^ T cells still contained abundant α2,6-sialylated sugar structures (**Figure 2B**). Moreover, sialylation of different leucocyte populations in blood, spleen, and BM of ST6Gal1^−/−^/μMT: PepBoy mice were analyzed by flow cytometry using SNA staining (gating strategy, see **Supplementary Figure 1A**). While T cells were sialylated to a comparable level in ST6Gal1^−/−^/μMT: PepBoy, C57BL/6, and BL6/μMT: PepBoy mice, different B-cell subpopulations of blood, spleen, and BM were completely lacking α2,6-linked sialic acid residues (**Figure 2C**). As expected, pro B cells were still positive for SNA as μMT mice have an arrest at the pro- to pre-B-cell stage and thus still have SNA^+^ pro B cells. Sialylation of other leucocyte populations like neutrophils, eosinophils, and monocytes was also similar to controls (**Supplementary Figure 1B**).

### 2.3 A fraction of antibody-secreting B-cell subsets is irradiation resistant and contributes to serum IgG sialylation in BM chimeric mice

As BM recipient PepBoy mice express the allelic marker CD45.1 and donor mice express CD45.2 (see **Figure 2A**), reconstitution efficiency could be assessed in different organs like blood, spleen and BM (**Supplementary Figure 2**). Consistent with previous reports, 10%–20% of CD4^+^ T cells in all three organs and 15%–20% of CD8^+^ T cells in the spleen and BM were still of recipient origin (28–30). Unexpectedly, however, also 5%–10% recipient-derived CD138^+^ TACI^+^ plasma blasts and plasma cells could be detected in BM chimeric animal cohorts.

**Figure 3:**
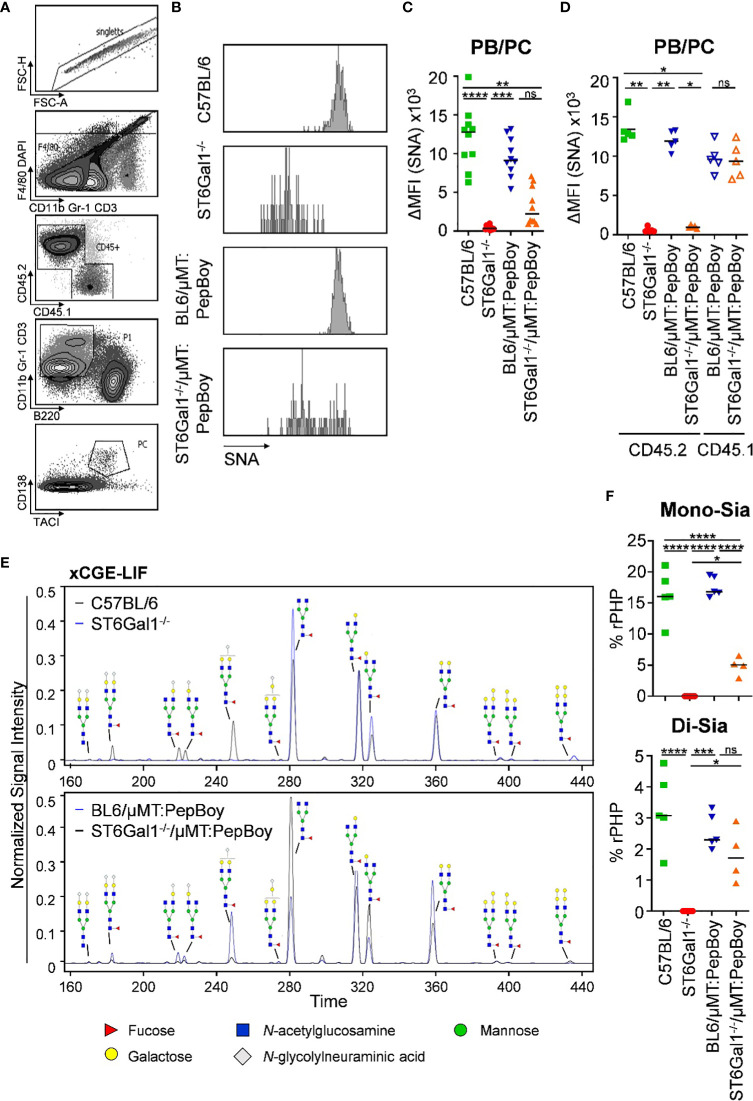
Detection of SNA^+^ plasma blasts/plasma cells and sialic acid residues in serum IgG of ST6Gal1^−/−^/µMT: PepBoy mice. **(A, B)** Shown are **(A)** the gating strategy and **(B)** representative histograms for SNA (*Sambucus nigra* agglutinin) expression on plasma cells in the indicated mouse cohorts. **(C, D)** Depicted are delta median fluorescence intensities (ΔMFI) of SNA expression on plasma blasts/plasma cells (PB/PC) as determined by flow cytometry on **(C)** total or **(D)** bone marrow donor/recipient specific PB/PC pool. **(E, F)** Determination of serum IgG glycosylation via multiplexed capillary gel electrophoresis with laser-induced fluorescence (xCGE-LIF). Shown are **(E)** representative electropherograms and **(F)** the quantification of mono- (Mono-Sia) and di-sialylated (Di-Sia) sugar residues. %rPHP: relative peak height proportions. *p < 0.05; **p < 0.01; ***p < 0.001; ****p < 0.0001; ns, not significant.

Furthermore, although B-cell subpopulations beginning from pre-B cells were all SNA negative (**Figure 2C**), α2,6-linked sialic acid residues on splenic CD138^+^ TACI^+^ plasma blasts and plasma cells (**Figure 3A**) were not significantly decreased in ST6Gal1^−/−^/μMT: PepBoy mice showing two peaks in the histogram for SNA corresponding to either WT or ST6Gal1^−/−^ mice (**Figures 3B, C**). When separating CD45.1- and CD45.2-positive plasma blasts and plasma cells, CD45.2^+^ cells were not sialylated while CD45.1^+^ residual recipient plasma blasts and plasma cells were sialylated to a level comparable to WT controls (**Figure 3D**). Thus, our data suggest that a small plasma cell pool, which existed in BM recipient PepBoy mice before irradiation, survived the irradiation regimen. Consistent with the presence of ST6Gal1-sufficient plasma blasts and plasma cells, clearly detectable peaks corresponding to sialylated IgG species could be detected in the serum of ST6Gal1^−/−^/μMT: PepBoy mice via xCGE-LIF (multiplexed capillary gel electrophoresis with laser-induced fluorescence) analysis (**Figure 3E**). While mono-sialylated species, carrying only one α2,6-linked sialic acid residue, constituted 5% of all sugar species in ST6Gal1^−/−^/μMT: PepBoy mice, in WT controls, this sugar species was threefold more abundant (15% of all sugar species) (**Figure 3F**). In contrast, di-sialylated sugar moieties were almost comparable to controls (C57BL/6: 3%; BL6/μMT: PepBoy: 2.2%; ST6Gal1^−/−^/μMT: PepBoy: 1.7%).

Very small unannotated glycan peaks within sialylated glycan species in total ST6Gal1^−/−^ mice could be identified as mainly α2,3-linked sialic acid residues, as treatment with α2,3-specific or α2,3/6/8/9-specific sialidases removed all glycan peaks correlating to these sialic acid residues (**Supplementary Figure 3**).

In summary, although all BM donor-derived B-cell subpopulations were completely lacking α2,6-linked sialic acids in ST6Gal1^−/−^/μMT: PepBoy mice, a small subset of irradiation-resistant SNA^+^ recipient plasma blasts and plasma cells contributed to serum IgG sialylation.

### 2.4 Lack of sialic acid residues leads to increased amounts of α-galactosylated glycan traits

**Figure 4:**
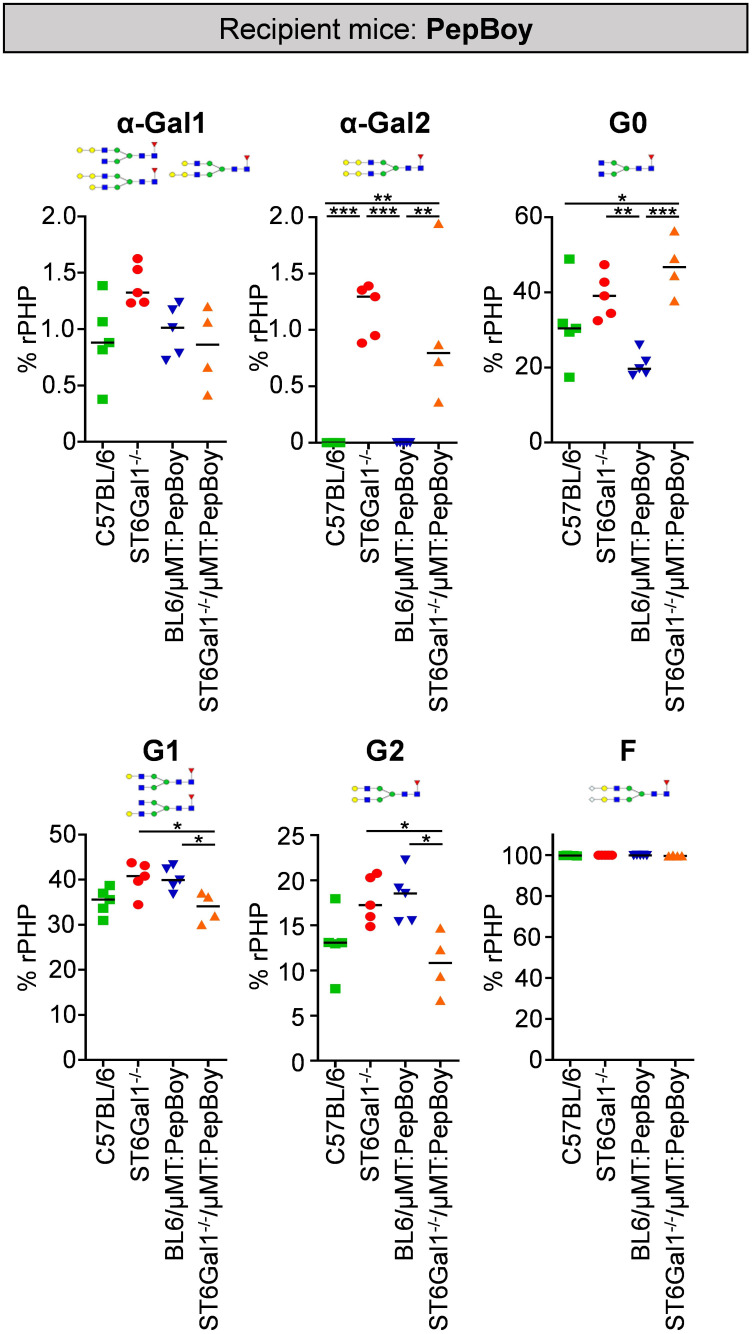
Glycan analysis of serum IgG in ST6Gal1^−/−^/µMT: PepBoy mice reveals increased amounts of α-galactosylated glycan traits. Serum IgG was purified via protein G and analyzed using multiplexed capillary gel electrophoresis with laser-induced fluorescence (xCGE-LIF). Depicted is the quantification of mono-α-galactosylated (α-Gal1), di-α-galactosylated (α-Gal2), agalactosylated (G0), mono-galactosylated (G1), di-galactosylated (G2), and fucosylated (F) sugar residues. Schematic glycan structures for each trait as well as an exemplary structure for fucosylated glycan residues are depicted. %rPHP: relative peak height proportions. *p < 0.05; **p < 0.01; ***p < 0.001.

To identify changes in IgG glycosylation in ST6Gal1^−/−^ mice beyond sialylation, different IgG glycan structures were quantified via xCGE-LIF analysis. As shown in **Figure 4**, especially di-α-galactosylated glycan structures carrying a terminal α1,3-linked galactose residues attached to beta-linked galactoses were significantly elevated while being completely absent in controls. This is probably due to sialic acid residues being naturally absent in ST6Gal1-deficient mice providing more substrate for enzymes adding α1,3-linked galactose residues. Moreover, glycan structures completely lacking galactose and sialic acids (G0) were more than twofold increased in ST6Gal1^−/−^/μMT: PepBoy mice, while mono- (G1) or di-galactosylated (G2) structures were slightly reduced. Thus, the lack of ST6Gal1 seems to lead to a completely altered IgG glycan repertoire.

### 2.5 Serum IgG sialylation is absent in BM recipients lacking pre-existing plasma cells

**Figure 5:**
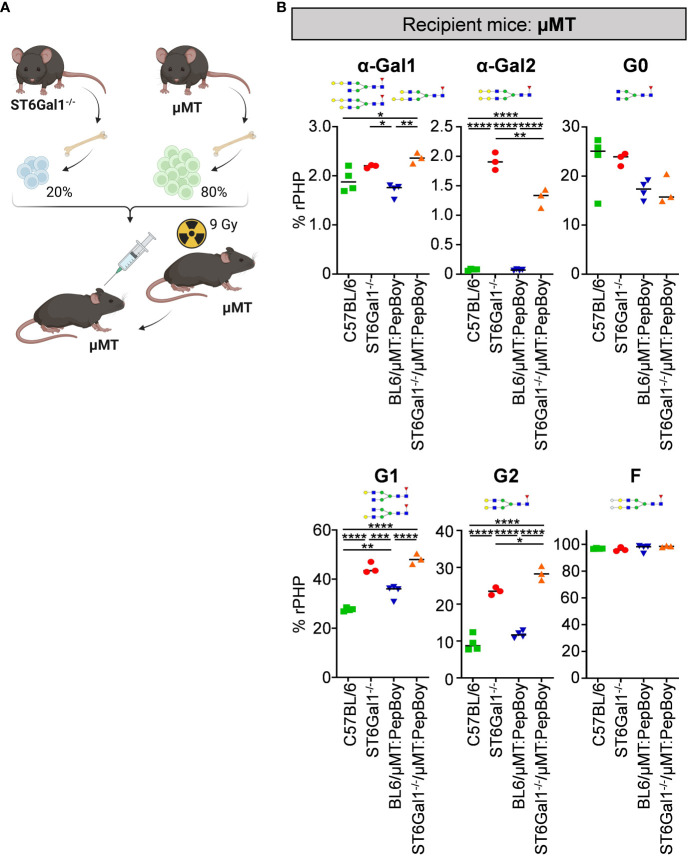
Analysis of non-sialylated IgG glycovariants in the serum of ST6Gal1^−/−^/µMT:µMT mice. **(A)** Schematic overview of the experimental strategy. **(B)** Shown is the quantification of mono-α-galactosylated (α-Gal1), di-α-galactosylated (α-Gal2), agalactosylated (G0), mono-galactosylated (G1), di-galactosylated (G2), and fucosylated (F) sugar residues as determined by multiplexed capillary gel electrophoresis with laser-induced fluorescence (xCGE-LIF). Schematic glycan structures for each trait as well as an exemplary structure for fucosylated glycan residues are depicted. %rPHP: relative peak height proportions. *p < 0.05; **p < 0.01; ***p < 0.001; ****p < 0.0001.

As our previous results emphasize that plasma cells/plasma blast subsets in wild-type BM recipients can survive irradiation, we adapted the BM chimeric model by using μMT mice not only as donor but also as recipient mice. Thus, we sublethally irradiated B cell-deficient μMT mice and reconstituted them with 20% BM of ST6Gal1^−/−^ mice and 80% BM of ST6Gal1^+/+^ µMT BM donor mice (ST6Gal1^−/−^/μMT:μMT) (**Figure 5A**). As ST6Gal1^−/−^/μMT: PepBoy mice showed elevated amounts of G0 glycan traits while G1 or G2 were significantly reduced, we also checked for general changes in IgG glycosylation in ST6Gal1^−/−^/μMT:μMT mice (**Figure 5B**). Again, we found significantly heightened levels of mono- and di-α-galactosylated glycan structures in mice lacking α2,6-linked sialic acids. Interestingly, however, agalactosylated glycan traits were not significantly altered and mono- (G1) and di-galactosylated (G2) sugar residues were increased in our revised BM chimeric mouse model. Thus, sufficient amounts of possible acceptor sites for B-cell extrinsic IgG sialylation were present in this mouse model specifically lacking ST6Gal1 in B cells.

**Figure 6:**
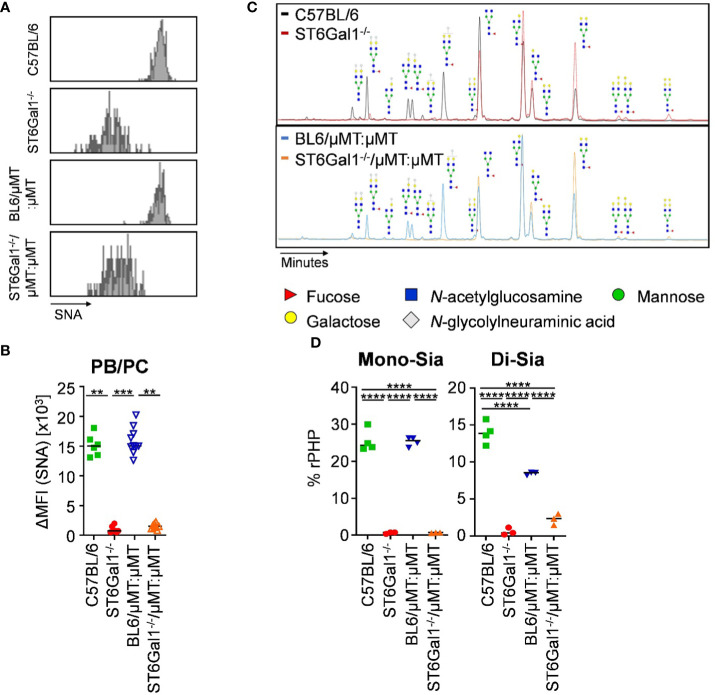
IgG sialylation *in vivo* occurs in B cells pre-antibody secretion. Plasma blasts/plasma cells in the spleen of ST6Gal1^−/−^/μMT:μMT mice were analyzed 8 to 10 weeks after reconstitution. **(A, B)** Shown are **(A)** representative histograms and **(B)** delta median fluorescence intensities (ΔMFI) of SNA (S*ambucus nigra agglutinin*) staining of plasma blasts/plasma cells (PB/PC) in the indicated mouse cohorts as determined by flow cytometry. **(C, D)** Shown are **(C)** representative electropherograms and **(D)** the quantification of mono- (Mono-Sia) and di-sialylated (Di-Sia) sugar residues on purified serum IgG via multiplexed capillary gel electrophoresis with laser-induced fluorescence (xCGE-LIF). Depicted are relative peak height proportions (%rPHP). **p < 0.01; ***p < 0.001; ****p < 0.0001.

As before, we analyzed the level of α2,6-linked sialic acid residues on plasma cells and on serum IgG of ST6Gal1^−/−^/μMT:μMT mice. In contrast to the previous chimeric mice using wild-type mice as recipients, splenic CD138^+^ TACI^+^ plasma blasts and plasma cells of ST6Gal1^−/−^/μMT:μMT mice consisted of a single SNA^-^ population (**Figure 6A**) with delta median fluorescence intensities for SNA being comparable to total ST6Gal1^−/−^ mice (**Figure 6B**). Moreover, analysis of serum IgG glycosylation via xCGE-LIF analysis confirmed the lack of virtually all sialylated glycan species with mono- and di-sialylated species in ST6Gal1^−/−^/μMT:μMT mice being comparable to total ST6Gal1^−/−^ mice (**Figures 6C, D**).

Taken together, our results demonstrate that IgG Fc sialylation almost exclusively occurs in plasma blasts/plasma cells pre-IgG secretion into the serum. Moreover, ST6Gal1 is critical for the normal development of late B-cell differentiation stages including germinal center B cells, plasma blasts, and plasma cells.

## 3 Discussion

Glycosylation of both the IgG F(ab) and the Fc domain plays a critical role in modulating the antigen-binding or downstream effector responses triggered by IgG, respectively. With respect to glycosylation of the IgG Fc domain, terminal sialic acid residues were shown to dampen pro-inflammatory and induce anti-inflammatory activities of the IgG molecule. Thus, a major effort in developing therapeutic antibodies is directed toward ensuring a specific glycosylation status of the therapeutic antibody. For example, cytotoxic antibodies aiming at depletion of malignant or autoreactive cells, need to interact optimally with cellular FcγRs and should have a glycosylation profile low in terminal sialic acid residues. As it was assumed that glycosylation of proteins, including IgG, occurs almost exclusively within cells, the glycosylation status of injected recombinant antibodies should remain rather stable. More recent studies have challenged this view, however, and suggest that terminal sialic acid residues are added to IgG antibodies predominantly after secretion from plasma blasts and plasma cells (22, 31–33). This raises the major concern that therapeutic antibodies injected into patients with autoimmune disease or cancer may change their glycosylation profile after administration. In the worst case, this may reduce their therapeutic activity over time or even turn pro-inflammatory effector functions into anti-inflammatory activities. We have recently addressed this concern by injecting antibodies with low or absent sialylation into mice and following their sialylation level during the next 6 days after injection (34). Surprisingly, we observed that less than 1% of the injected IgG molecules acquired terminal sialic acid residues. More importantly, the addition of sialic acid residues selectively occurred if the injected IgG molecules contained an accessible F(ab)-linked sugar moiety. This would indicate that the majority of therapeutic antibodies, which do not contain a F(ab)-linked sugar domain, will not change their glycosylation pattern and thus effector function upon administration. Indeed, we did not observe an altered activity of the CD20-specific antibody rituximab, which we isolated after injection and re-injected again to test for altered effector functions (34). However, it remained possible that a major change in *de novo* sialylation of serum antibodies may occur at later time points. Thus, the aim of our current study was to understand in general if and to what extent IgG sialylation occurs upon secretion of IgG from plasma blasts and plasma cells. As previous studies, including our own, suggested that B cells lacking ST6Gal1 have a defect in late B-cell development, resulting in strongly reduced amounts of germinal center B cells, plasma blasts, and plasma cells (21, 26, 27), we wanted to use a model system ensuring that all B cells indeed lack ST6Gal1 expression. Many Cre-deleter mouse strains, including CD19-Cre mice, are well known to delete target genes very effectively but not entirely in all B cells, which may at least be one explanation why previous studies observed serum IgG sialylation despite an almost complete deletion of ST6Gal1 (24, 25). For example, despite a clear reduction of sialylated CD19^+^ B cells in CD19-Cre^+/−^ ST6Gal1^fl/fl^ mice, almost half of BM CD138^+^ B220^low^ plasma cells showed an SNA staining comparable to plasma cells from ST6Gal1-sufficient mice in the initial publication by Jones et al. (22), indicating incomplete deletion of ST6Gal1 gene, resulting in the expansion of SNA^+^ antibody producing plasma cells, consistent with our observations in this study and as highlighted more recently (35). To circumvent this issue, we turned to a BM chimeric model in which all B cells derive from a total ST6Gal1 knockout mouse, thus ensuring a complete deletion of ST6Gal1 in all B cells. To achieve a B cell-specific knockout, we generated BM chimeric mice by injecting a mixture of 20% BM of ST6Gal1^−/−^ mice and 80% BM of B cell-deficient but ST6Gal1-sufficient µMT mice (all BM donors mice are CD45.2^+^) into irradiated CD45.1^+^ ST6Gal1-sufficient PepBoy mice (23). Of note, while, as expected, BM chimeric animals lacked ST6Gal1 expression specifically in B cells, almost comparable levels of mono- and especially di-sialylated IgG glycan species could be observed in serum IgG. A re-analysis of the origin of plasma blasts and plasma cells in these animals demonstrated that despite a lethal irradiation regimen, BM recipient-derived CD45.1^+^ plasma blasts/plasma cells were present in these mice. Indeed, previous studies have observed this low radiation sensitivity, especially of long-lived plasma cells before (36, 37).

To solve this issue, we further adopted our *in vivo* model system by using B cell-deficient µMT mice as recipient mice ensuring that all mature B-cell populations including plasma blasts and plasma cells can only develop from the injected ST6Gal1^−/−^ BM. Indeed, this model led to a full deletion of sialic acid residues on plasma blasts/plasma cells. In a similar manner, the level of serum IgG mono-sialylation dropped to the level observed in total ST6Gal1^−/−^ mice and also the level of di-sialylated IgG species was dramatically reduced but remained slightly higher compared to total ST6Gal1^−/−^ controls. We hypothesize that this minimal level of di-sialylated IgG species may indeed reflect B cell-extrinsic IgG sialylation most likely at the more easily accessible Fab-fragment-associated sugar domains, consistent with our previous observation (34, 35).

In summary, our study emphasizes that IgG Fc-linked sugar domains including terminal sialylation are added in plasma cells/plasma blasts before secretion and are not subject to sialylation post-IgG secretion. Thus, *in vivo*, no alterations in therapeutic IgG glycosylation occur due to B cell-extrinsic *de novo* sialylation processes. We cannot fully rule out, however, that a minimal level of *de novo* sialylation occurs at the IgG F(ab) fragment. To what extent these small changes in di-sialylated sugar structures on IgG modulate antigen recognition/IgG specificity remains to be elucidated.

## 4 Materials and methods

### 4.1 Mice

C57BL/6 mice were bought from Janvier or bred in-house, and μMT mice were obtained from The Jackson Laboratory. ST6Gal1^−/−^ mice were kindly provided by Prof. Dr. rer. nat. Lars Nitschke (FAU Erlangen, Germany). B6.SJL-PtprcaPepcb/BoyJ (PepBoy) mice were bred in-house. Before an initial experiment, all mice were kept in the animal facilities of Friedrich-Alexander-University Erlangen-Nuremberg under specific pathogen-free conditions in individually ventilated cages, in accordance with the guidelines of the NIH and the legal requirements of Germany and the USA. All animal experiments conducted in the animal facility of the FAU were approved by the government of lower Franconia.

### 4.2 Generation of bone marrow chimeric mice

Eight- to ten-week-old μMT or PepBoy mice were sublethally irradiated with 9 Gy (γ-irradiation via a cesium source) and reconstituted with a mixture of 80% BM of μMT and 20% BM of ST6Gal1^−/−^ or C57BL/6 mice 8 to 14 h after irradiation. Chimeras were left to fully reconstitute their peripheral lymphoid system over at least 8 weeks before further use.

### 4.3 Fluorescence-activated cell sorting analysis

Murine blood was obtained from the retroorbital plexus, and spleen and BM cells were harvested and flushed trough a 70-µm cell strainer to receive single-cell suspensions. Erythrocytes were lysed with water lysis. To reduce unspecific binding to Fc receptors, cells were incubated on ice for at least 10 min with Fc block (clone 2.4G2, 10 µg/mL and 9E9, 10 µg/mL). Cells were washed and incubated on ice for at least 15 min with combinations of antibodies against the following antigens: CD45, TCRβ, Gr-1, CD11b, Ly6G, NKp46, B220, IgM, IgD, CD23, CD21/35, CD95, and CD43 (all from BioLegend). FITC-conjugated SNA was purchased from Vector Laboratories. Analysis was restricted to viable cells, which were identified by exclusion of cells positive for the nucleic acid binding dye 4′6-diamino-2-phenylindol (DAPI). Experiments were acquired on a FACS Canto II (BD) and analyzed using FACS Diva software (BD).

### 4.4 Immunofluorescence microscopy

Five-micrometer sections of frozen tissue were air-dried overnight, followed by fixation in acetone. Slides were blocked with 5% goat serum in PBS and stained with A647-conjugated anti-CD3ϵ (BioLegend), PE-conjugated anti-B220 (BD), and FITC-conjugated SNA (Vector Laboratories). After incubation, the excess of fluorescent dye was removed by multiple washing steps with PBS, and slides were mounted using Fluoromount imaging medium (Sigma) and analyzed on a Zeiss Axio Observer microscope with the Zen blue 2.5 software.

### 4.5 Glycan analysis

#### 4.5.1 IgG isolation

For PepBoy recipient mice, the IgG was isolated using protein G monolithic plates (BIA Separations, Ajdovščina, Slovenia) as described previously (38). Briefly, 100 to 400 µL of serum was diluted at a ratio of 1:7 with 1× PBS, pH 7.4, and filtered through a 0.45-μm GHP filter plate (Pall Corporation, Ann Arbor, MI, USA). After filtration, serum samples were applied to the protein G plate and instantly washed with 1× PBS, pH 7.4, to remove unbound proteins. IgGs were eluted with 1 mL of 0.1 M formic acid (Merck, Darmstadt, Germany) and neutralized with 1 M ammonium bicarbonate (Merck, Darmstadt, Germany).

For µMT recipient mice, the volume of 40 μL of Protein G beads slurry (50% slurry; Protein G Agarose Fast Flow, Merck Millipore, MA, USA) was dispensed into each well of an Orochem filter plate (Orochem Technologies Inc., USA) and washed three times with 200 μL of 1× PBS (pH 7.4) on a vacuum manifold. To isolate IgG, 50 μL of mouse serum was diluted at a ratio of 1:7 with 1× PBS, pH 7.4, and filtered through a 0.45-μm GHP filter plate (Pall Corporation, Ann Arbor, MI, USA). Filtered serum samples were transferred to the Orochem plate containing Protein G beads and incubated for 3 h at room temperature. After incubation, each well was washed three times with 300 μL followed by three washes with 300 μL of ultra-pure water to remove unbound antibodies and other contaminants. For elution of IgG, 100 μL of 0.1 M formic acid (pH 2.5) was added to each well of the Orochem filter and incubated for 15 min at RT. IgGs were collected in a clean PCR plate and neutralized with 17 μL of 1 M ammonium bicarbonate (Merck, Darmstadt, Germany).

#### 4.5.2 Sample preparation for xCGE-LIF analysis of glycans

##### 4.5.2.1 Methanol desalting

Volumes of IgG eluates corresponding from 3 to 10 μg of IgG were dried in a vacuum concentrator and desalted. To precipitate and desalt isolated IgG, 800 μL of cold methanol was added to each well of a 2-mL collection plate, resuspended, and centrifuged for 15 min at 2,000×*g*. Afterwards, 780 μL of supernatant was carefully removed into a waste. The previous steps were repeated one more time and precipitated IgGs were dried in a vacuum concentrator.

##### 4.5.2.2 N-glycan release

The dried and desalted samples were resuspended in 3 μL of 1.66× PBS and 4 μL of 0.5% SDS (w/v) (Invitrogen, Carlsbad, CA, USA) and denatured by incubation at 65°C for 10 min. After incubation, 2 μL of 4% Igepal-CA630 (Sigma-Aldrich, St. Louis, MO, USA) was added to the samples and incubated on a shaker for 15 min. After shaking, 1.2 U of PNGase F (Promega, Madison, WI, USA) in 1 μL of 5× PBS was added and incubated for 3 h at 37°C for N-glycan release. Samples were dried in a vacuum concentrator afterwards (39).

##### 4.5.2.3 Glycan labeling and HILIC-SPE

Dried samples were labeled with aminopyrene-1,3,6-trisulfonic acid (APTS, Sigma-Aldrich, St. Louis, MO, USA) and cleaned by HILIC-SPE on BioGel P10 (Bio-Rad, Hercules, CA, USA) in a 96-well format as described previously (39, 40).

#### 4.5.3 Sialidase digestion

α2–3,6,8,9 Sialidase (P0722L) and α2–3 Sialidase (P0722L) were obtained from NEB. For removal of sialic acids, 8 μL of APTS-labeled mouse IgG N-glycans (equivalent of approximately 2.5 μg of IgG) was incubated with 1 μL of 10× Glyco buffer and 1 μL of enzyme for 4 h at 37°C. The reactivity and specificity of each enzyme were confirmed by digestion of human total plasma APTS N-glycans for α2–3 Sialidase and human IgG APTS N-glycans for α2–3,6,8,9 Sialidase (positive controls).

#### 4.5.4 Multiplexed capillary gel electrophoresis with laser-induced fluorescence

The N-glycosylation pattern of IgG purified from the serum of PepBoy recipient mice was assessed by the xCGE-LIF. The reaction mixture consisted of 3 µL of N-glycan post-cleanup eluate, 1 µL of GeneScan 500 LIZ Size Standard (Applied Biosystems, Foster City, CA, USA; 1:50 dilution in Hi-Di Formamide), and 6 µL of Hi-Di Formamide (Applied Biosystems, Foster City, CA, USA) pipetted into a MicroAmp Optical 96-well Reaction Plate (Applied Biosystems), sealed with 96-well plate septa (Applied Biosystems), and briefly centrifuged to avoid air bubbles at the bottom of the wells. Analysis was performed on a 3130 Genetic Analyzer (Applied Biosystems), equipped with a 50-cm four-capillary array filled with POP-7 polymer (Applied Biosystems). Oven temperature was set at 30°C. Electrokinetic sample injection was performed at 7.5 to 15 kV for 5 or 10 s depending on the signal intensity; samples were analyzed with a running voltage of 15 kV and a run time of 3400 s. Raw data files were converted to.xml file format using DataFileConverter (Applied Biosystems) and analyzed using the glycan analysis tool glyXtool™ (glyXera, Magdeburg, Germany). GlyXtool™ software was used for structural identification by patented migration time normalization to an internal standard and N-glycan database-driven peak annotation, for data comparison and for integration of normalized peak heights (40, 41). In total, 13 IgG N-glycan peaks were identified and relatively quantified in each electropherogram. In other words, the amount of glycans in each peak was expressed as relative peak height proportions (rPHP, %).

N-glycosylation analysis of IgG purified from the serum of µMT recipient mice was conducted by xCGE-LIF as well but with several modifications included. The reaction mixture consisted of 2 µL of N-glycan post-cleanup eluate and 8 µL of Hi-Di Formamide (Applied Biosystems). Analysis was performed on a 3500 Genetic Analyzer (Applied Biosystems), equipped with a 50-cm eight-capillary array filled with POP-7 polymer (Applied Biosystems). Oven temperature was set at 60°C. Electrokinetic sample injection was performed at 15 kV for up to 9 s, depending on the signal intensity. Samples were analyzed with a running voltage of 19.5 kV and a run time of 1,800 s. Obtained electropherograms were manually integrated in 13 N-glycan peaks using Empower 3 software and relatively quantified as described in the text above.

### 4.6 Quantification and statistical analysis

Data were analyzed and plotted with GraphPad Prism 8.0 software (GraphPad Software Inc., San Diego, CA). Outliers were removed by the ROUT method. Normal distribution was tested with a Shapiro–Wilk normality test. For two groups, normally distributed data were analyzed by unpaired *t*-test and non-normally distributed data were analyzed by Mann–Whitney *U*-test. For more than two groups, normally distributed data were analyzed by ordinary one-way ANOVA followed by Holm–Sidak’s multiple comparison test. Non-parametric distributed data were tested using Kruskal–Wallis test with Dunn’s *post-hoc* test. *p* < 0.05 was considered significant.

## Data availability statement

The original contributions presented in the study are included in the article/**Supplementary Material**. Further inquiries can be directed to the corresponding authors.

## Ethics statement

The animal study was approved by Regierung von Unterfranken, Würzburg, Germany. The study was conducted in accordance with the local legislation and institutional requirements.

## Author contributions

AW: Conceptualization, Investigation, Project administration, Writing – original draft, Funding acquisition. MH: Investigation, Writing – review & editing. OZ: Writing – review & editing. GL: Writing – review & editing. AL: Supervision, Writing – review & editing. LN: Writing – review & editing, Funding acquisition. FN: Conceptualization, Supervision, Writing – original draft, Writing – review & editing, Funding acquisition.
