# Supplementary material for: IgG sialylation occurs in B cells pre antibody secretion
Source: Front Immunol. 2024 May 17;15:1402000. doi: 10.3389/fimmu.2024.1402000 (PMC11140079; doi:10.3389/fimmu.2024.1402000)
Supplement: Supplementary file 1 [file DataSheet_1.docx]

Supplementary Material


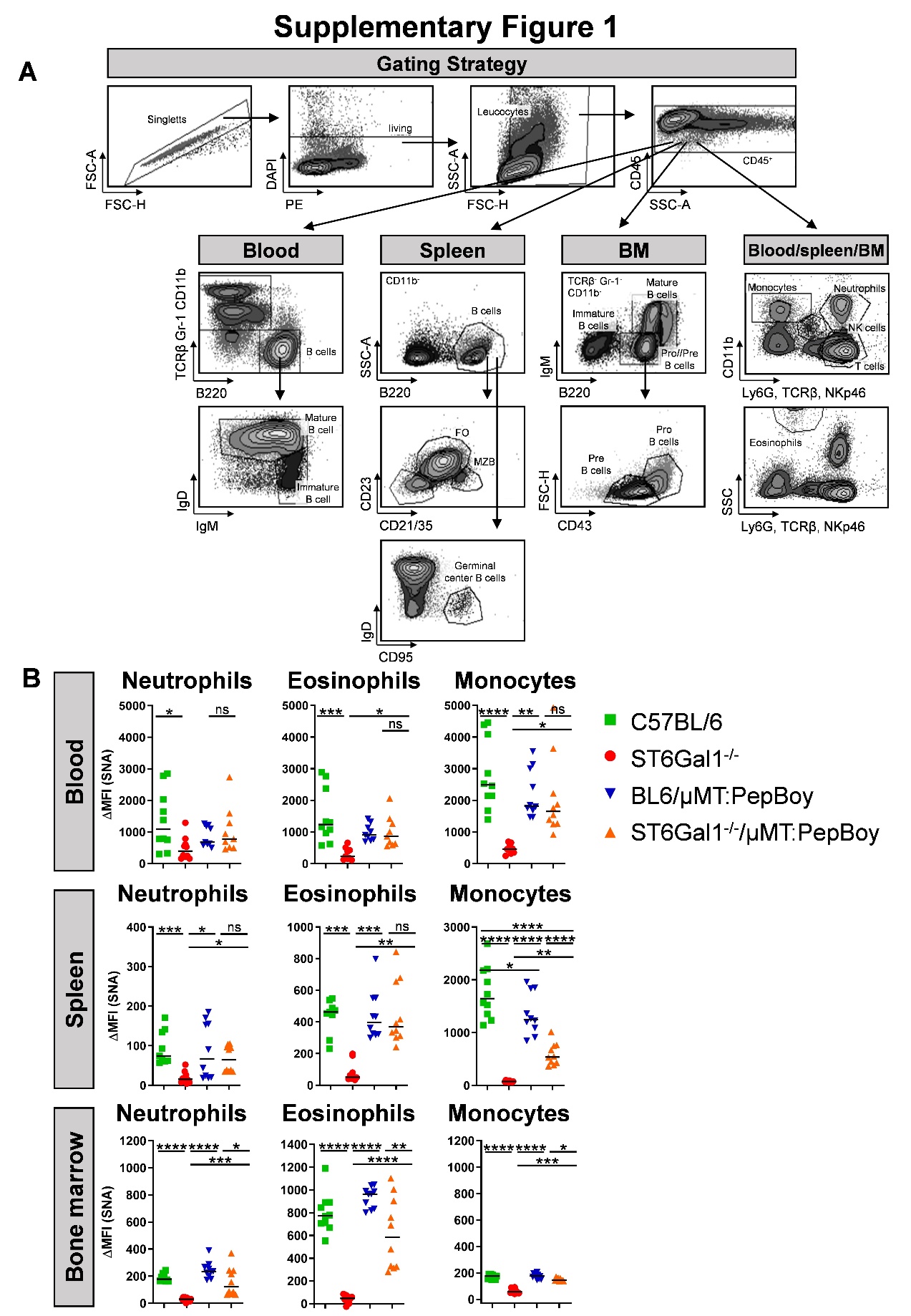


**Supplementary Figure 1: Gating strategy and α2,6 linked sialic acid residues on neutrophils, eosinophils and monocytes.**

(A) Shown is an exemplary gating strategy to identify the indicated immune cell subsets in blood, spleen and bone marrow (BM) via flow cytometry. (B) Depicted is the quantification of the delta median fluorescence intensities (ΔMFI) of SNA (*sambucus nigra agglutinin*) staining on the indicated immune cell subsets in the respective organs as determined by flow cytometry. **p < 0.05; ** p < 0.01; *** p < 0.001; **** p < 0.0001.*

**
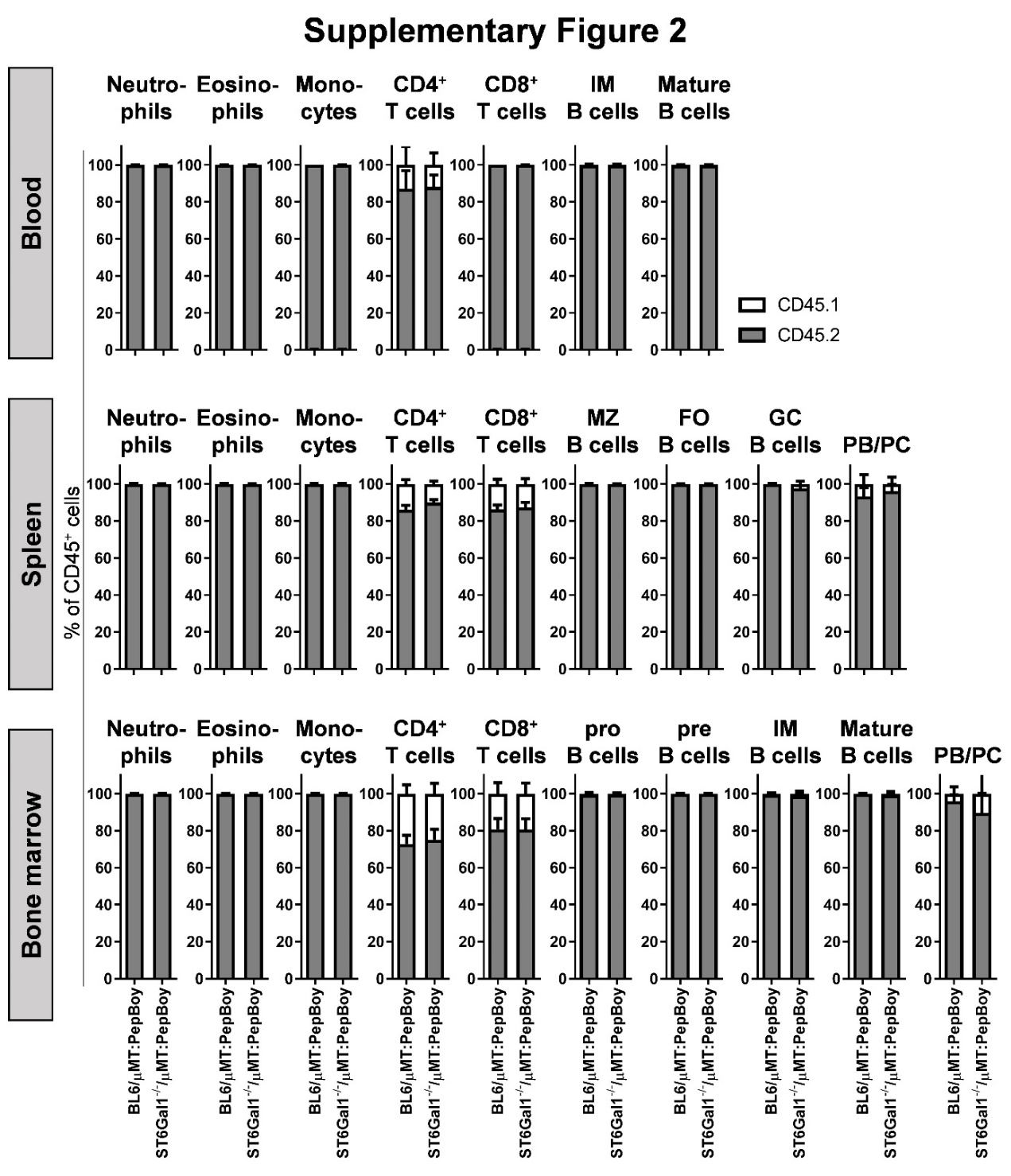
**



**Supplementary Figure 2: Immune cell subset specific chimerism after bone marrow transplantation**

Shown is the relative abundance of CD45.1^+^ or CD45.2^+^ cells within all CD45^+^ cells of the indicated cell population in the different organs of the indicated bone marrow chimeric mouse strains eight weeks after bone marrow transplantation as determined by flow cytometry. IM = immature; MZ = marginal zone; FO = follicular; GC = germinal centre; PB/PC = plasma blast / plasma cell.


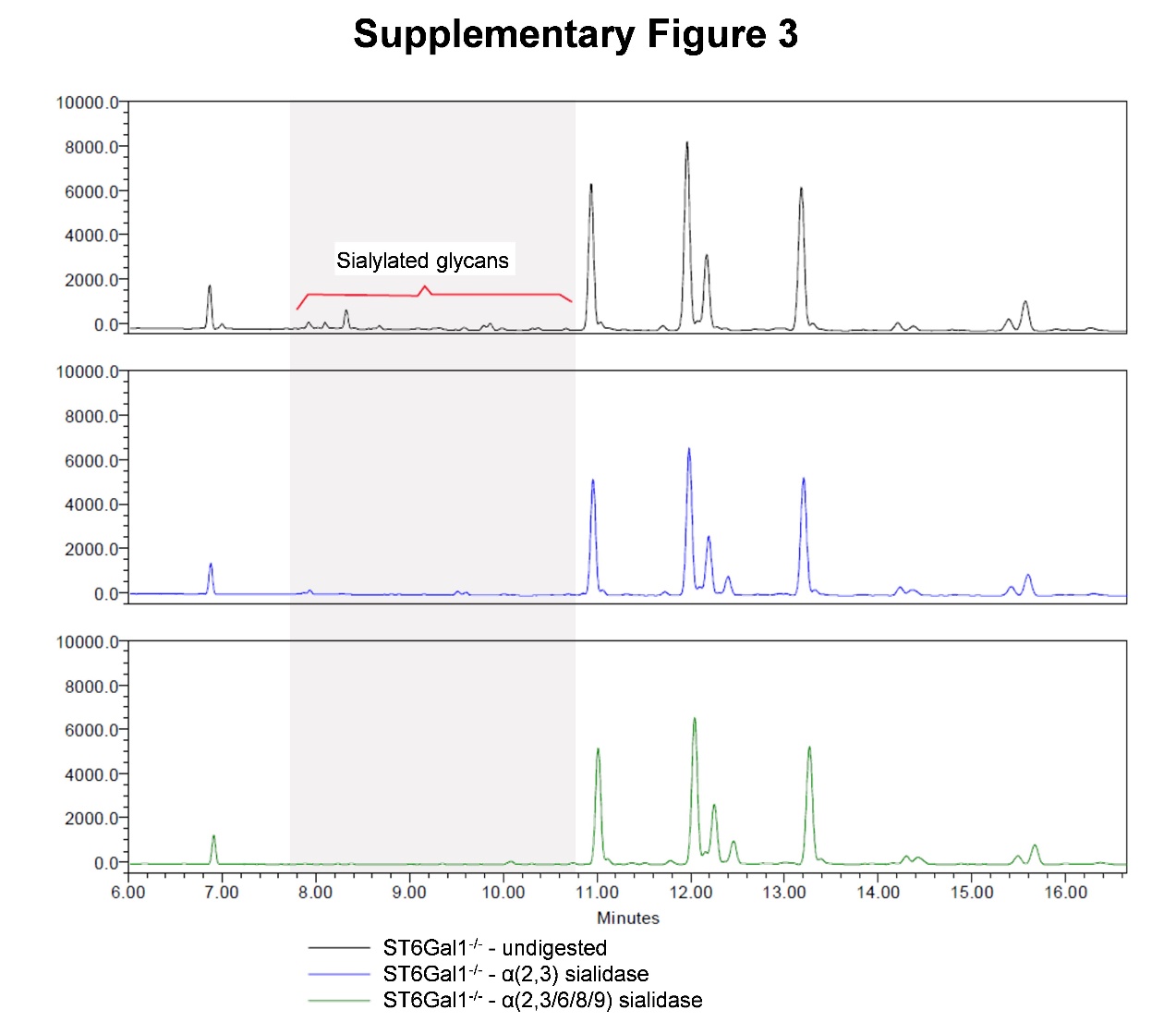


**Supplementary Figure 3: Sialidase digestions of serum IgG of total ST6Gal1^-/-^ mice**

Serum IgG preparations of total ST6Gal1^-/-^ mice were left undigested (black), digested with an alpha-2,3-specific sialidase (α(2,3) sialidase; blue) or with an alpha-2,3/6/8/9-specific sialidase (α(2,3/6/8/9) sialidase; green) and glycans analysed by xCGE-LIF analysis. Shown are representative electropherograms of different preparations with sialylated glycan species highlighted in lighter grey.
